# Crystal structure of *catena*-poly[[bis­(*N*-acethyl­thio­morpholine-κ*S*)copper(I)]-μ-iodido]

**DOI:** 10.1107/S2056989016019794

**Published:** 2017-01-01

**Authors:** Hojae Chiang, Tae Ho Kim, Hyunjin Park, Jineun Kim

**Affiliations:** a Department of Chemistry (BK21 plus) and Research Institute of Natural Sciences, Gyeongsang National University, Jinju 52828, Republic of Korea

**Keywords:** crystal structure, *N*-acetyl­thio­morpholine, coordination polymer, copper(I) iodide

## Abstract

The reaction of copper(I) iodide with *N*-acetyl­thio­morpholine (*L*, C_6_H_11_NOS) in aceto­nitrile results in a coordination polymer with composition [CuI(*L*)_2_]_*n*_. The Cu^I^ atom is coordinated by two S atoms and two I atoms, adopting a distorted tetra­hedral environment. The μ_2_-bridging mode of the I atoms gives rise to chains extending parallel to [010]. C—H⋯O hydrogen-bonding inter­actions between the chains lead to a three-dimensional network.

## Abstract 2

In the title compound, the Cu^I^ atom is coordinated by two S atoms and two I atoms in a distorted tetra­hedral mode.

## Chemical context   

Synthesis, structures and luminescence properties of copper(I) complexes involving CuI and thio­ethers as co-ligands have been studied extensively (Harvey & Knorr, 2010 ▸; Knorr *et al.*, 2010 ▸; Henline *et al.*, 2014 ▸). The tendency of copper(I) iodide to form aggregates often leads to short Cu—Cu bonds and intriguing diversities in the respective crystal structures (Peng *et al.*, 2010 ▸), comprising of [CuI]_*n*_ chains with split stair motifs (Moreno *et al.*, 1995 ▸; Blake *et al.*, 1999 ▸; Cariati *et al.*, 2002 ▸; Näther *et al.*, 2003 ▸; Thébault *et al.*, 2006 ▸), zigzag chains (Munakata *et al.*, 1997 ▸) or helical chains (Munakata *et al.*, 1997 ▸; Kang & Anson, 1995 ▸). Most of these structures include aromatic nitro­gen donor co-ligands. In this context we have studied the inter­action of *N*-acetyl­thio­morpholine with CuI to investigate the coordination behaviour of the copper(I) atom with the S donor atom of the *N*-acetyl­thio­morpholine co-ligand, because both are soft atoms in the sense of the HSAB concept. Although a number of copper(I) complexes with thio­ether ligands are known (Knorr *et al.*, 2010 ▸; Henline *et al.*, 2014 ▸), to the best of our knowledge, a [CuI]_*n*_ chain structure has not been reported until now. Herein, we report a copper(I) coordination polymer with a zigzag chain [CuI]_*n*_, resulting from the reaction of CuI with *N*-acetyl­thio­morpholine (*L*).
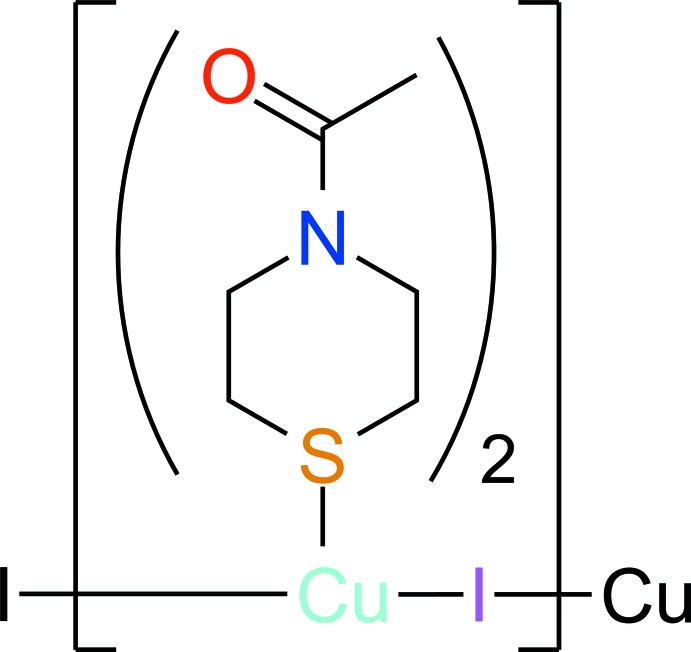


## Structural commentary   

**Figure 1:**
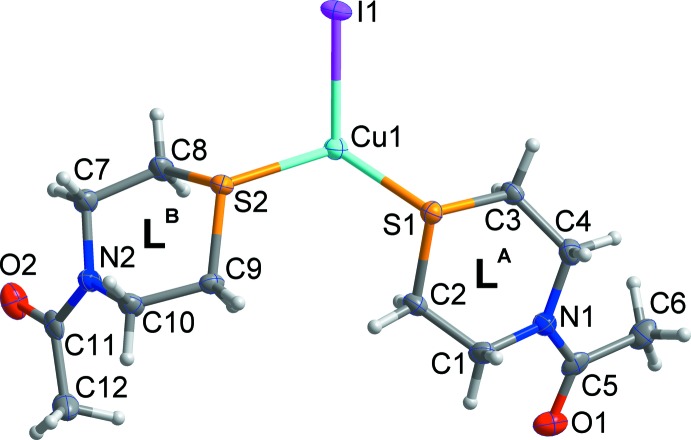
The asymmetric unit of the title compound, shown with displacement ellipsoids drawn at the 50% probability level. H atom are shown as small spheres of arbitrary radius.

**Figure 2:**
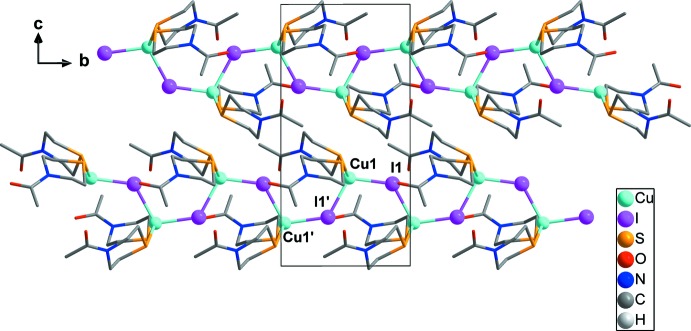
The polymeric chain structure in [CuI(*L*)_2_] formed through the μ_2_-bridging mode of the I atoms. All H atoms have been omitted for clarity.

The asymmetric unit of the title compound, [CuI(*L*)_2_]_*n*_, comprises of a copper(I) iodide moiety and two *N*-acetyl­thio­morpholine co-ligands (*L* ^A^ and *L* ^B^) and is shown in Fig. 1 ▸. The Cu^I^ atom has a slightly distorted tetra­hedral environment (Table 1 ▸). The two thio­morpholine rings have the stable chair conformation (Kang *et al.*, 2015 ▸). The dihedral angles between acetyl CCO and thio­morpholine CNC planes are 3.9 (4) and 6.6 (2)° for *L* ^A^ and *L* ^B^, respectively. The I atoms link neighboring Cu^I^ atoms in a *μ*_2_ *-*bridging mode into polymeric zigzag chains extending parallel to [010] (Fig. 2 ▸).

## Supra­molecular features   

**Figure 3:**
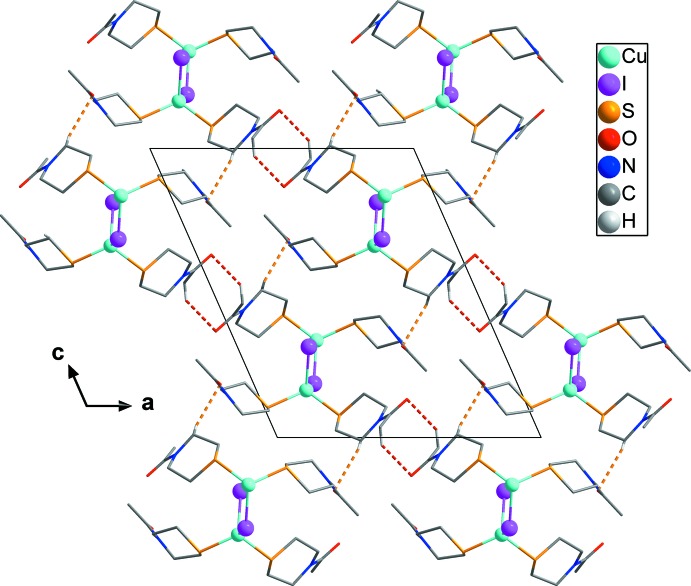
The crystal structure of [CuI(*L*)_2_] in a projection along [010]. C—H⋯O hydrogen bonds are shown as yellow and red dashed lines. H atoms not involved in inter­molecular inter­actions have been omitted for clarity.

As shown in Fig. 3 ▸, C10—H10*A*⋯ O1 hydrogen bonds (yellow dashed lines) between the thio­morpholine ring of *L* ^B^ and the carbonyl oxygen atoms of *L* ^A^ result in a layered network parallel to (101). Additional C12—H12*B*⋯O2 hydrogen bonds between methyl groups of *L* ^B^ ligands and carbonyl oxygen atoms of neighbouring *L* ^B^ ligands (red dashed lines) form cyclic centrosymmetric dimers of *N*-acetyl­thio­morpholines. The combination of the [CuI]_*n*_ chains and the two types of hydrogen-bonding inter­actions with additional C—H⋯O inter­actions (Table 2 ▸) leads to a three-dimensional network.

## Synthesis and crystallization   

Preparation of *N*-acetyl­thio­morpholine (*L*)

Thio­morpholine (1.03 g, 0.010 mol) and tri­ethyl­amine (1.03 g, 0.010 mol) in chloro­form (20 mL) were placed in a one-neck round-bottomed flask and kept at 273 K. Then, acetic anhydride (1.02 g, 0.010 mol) was added dropwise. The reactant mixture was stirred for approximately one day. The orange liquid product was purified by using short column chromatography (silica gel, 90% *n*-hexane and 10% ethyl acetate, *R_f_* = 0.28; yield 1.08 g, 74.5%). ^1^H NMR (300 MHz, CDCl_3_) / ppm: 3.860 (triplet, 2H, CH_2_-N), 3.719 (triplet, 2H, CH_2_-N), 2.614 (triplet, 2H, CH_2_-S), 2.597 (triplet, 2H, CH_2_-S), 2.086 (singlet, 3H, CH_3_); ^13^C NMR (300MHz, CDCl_3_) / ppm: 168.919 (C=O); 48.993, 43.972 (N—C); 27.248, 27.740 (S—C), 21.527(CH_3_)

Preparation of [CuI(*L*)_2_]_*n*_

An aceto­nitrile (2 mL) solution of *L* (0.08 g, 0.55 mmol) was allowed to mix with an aceto­nitrile (3 mL) solution of CuI (0.052 g, 0.27 mmol). The colorless precipitate was filtered and washed with diethyl ether/aceto­nitrile (3/1 *v*/*v*) solution (yield 0.116 g, 88.5%). Single crystals suitable for X-ray analysis were obtained by slow evaporation.

## Refinement   

Crystal data, data collection and structure refinement details are summarized in Table 3 ▸. All C-bound H atoms were positioned geometrically, with *d*(C—H) = 0.99 Å, *U*_iso_ = 1.2*U*_eq_(C) for methyl­ene, and *d*(C—H) = 0.98 Å, *U*_iso_ = 1.5*U*_eq_(C) for methyl groups.

## Supplementary Material

Crystal structure: contains datablock(s) I, New_Global_Publ_Block. DOI: 10.1107/S2056989016019794/wm5347sup1.cif

Structure factors: contains datablock(s) I. DOI: 10.1107/S2056989016019794/wm5347Isup2.hkl

CCDC reference: 1522053

Additional supporting information: crystallographic information; 3D view; checkCIF report

## Figures and Tables

**Table 1:** Selected geometric parameters (Å, °)

Cu1—S1	2.3012 (6)	Cu1—I1	2.6221 (3)
Cu1—S2	2.3064 (6)	Cu1—I1^i^	2.6476 (3)
			
S1—Cu1—S2	114.28 (2)	S2—Cu1—I1	101.246 (16)
S1—Cu1—I1	112.179 (17)	I1—Cu1—I1^i^	109.949 (9)

Symmetry code: (i) 
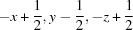
.

**Table 2:** Hydrogen-bond geometry (Å, °)

*D*—H⋯*A*	*D*—H	H⋯*A*	*D*⋯*A*	*D*—H⋯*A*
C4—H4*A*⋯O2^ii^	0.99	2.52	3.241 (3)	129
C6—H6*B*⋯O2^ii^	0.98	2.47	3.418 (3)	162
C10—H10*A*⋯O1^iii^	0.99	2.58	3.144 (3)	116
C12—H12*B*⋯O2^iv^	0.98	2.59	3.372 (3)	137

Symmetry codes: (ii) 
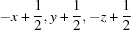
; (iii) 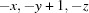; (iv) 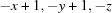.

**Table 3:** Experimental details

Crystal data	
Chemical formula	[CuI(C_6_H_11_NOS)_2_]
*M*_r_	480.87
Crystal system, space group	Monoclinic, *P*2_1_/*n*
Temperature (K)	173
*a*, *b*, *c* (Å)	14.1513 (4), 7.6557 (2), 16.9423 (4)
β (°)	113.805 (1)
*V* (Å^3^)	1679.34 (8)
*Z*	4
Radiation type	Mo *K*α
μ (mm^−1^)	3.39
Crystal size (mm)	0.40 × 0.10 × 0.02

Data collection	
Diffractometer	Bruker APEXII CCD
Absorption correction	Multi-scan (*SADABS*; Bruker, 2014 ▸)
*T*_min_, *T*_max_	0.518, 0.746
No. of measured, independent and observed [*I* > 2σ(*I*)] reflections	12664, 3306, 3020
*R*_int_	0.023
(sin θ/λ)_max_ (Å^−1^)	0.617

Refinement	
*R*[*F* ^2^ > 2σ(*F* ^2^)], *wR*(*F* ^2^), *S*	0.018, 0.045, 1.05
No. of reflections	3306
No. of parameters	183
H-atom treatment	H-atom parameters constrained
Δρ_max_, Δρ_min_ (e Å^−3^)	0.51, −0.37

Computer programs: *APEX2* and *SAINT* (Bruker, 2014 ▸), *SHELXS97* and *SHELXTL* (Sheldrick, 2008 ▸), *SHELXL2014* (Sheldrick, 2015 ▸), *DIAMOND* (Brandenburg, 2010 ▸) and *publCIF* (Westrip, 2010 ▸).

## supplementary crystallographic information

### Crystal data

**Table:**

[CuI(C_6_H_11_NOS)_2_]	*F*(000) = 952
*M**_r_* = 480.87	*D*_x_ = 1.902 Mg m^−^^3^
Monoclinic, *P*2_1_/*n*	Mo *K*α radiation, λ = 0.71073 Å
*a* = 14.1513 (4) Å	Cell parameters from 8258 reflections
*b* = 7.6557 (2) Å	θ = 2.4–27.4°
*c* = 16.9423 (4) Å	µ = 3.39 mm^−^^1^
β = 113.805 (1)°	*T* = 173 K
*V* = 1679.34 (8) Å^3^	Plate, colourless
*Z* = 4	0.40 × 0.10 × 0.02 mm

### Data collection

**Table:**

Bruker APEXII CCD diffractometer	3020 reflections with *I* > 2σ(*I*)
φ and ω scans	*R*_int_ = 0.023
Absorption correction: multi-scan (*SADABS*; Bruker, 2014)	θ_max_ = 26.0°, θ_min_ = 1.6°
*T*_min_ = 0.518, *T*_max_ = 0.746	*h* = −13→17
12664 measured reflections	*k* = −9→9
3306 independent reflections	*l* = −20→20

### Refinement

**Table:**

Refinement on *F*^2^	0 restraints
Least-squares matrix: full	Hydrogen site location: inferred from neighbouring sites
*R*[*F*^2^ > 2σ(*F*^2^)] = 0.018	H-atom parameters constrained
*wR*(*F*^2^) = 0.045	*w* = 1/[σ^2^(*F*_o_^2^) + (0.0221*P*)^2^ + 0.5521*P*] where *P* = (*F*_o_^2^ + 2*F*_c_^2^)/3
*S* = 1.05	(Δ/σ)_max_ = 0.003
3306 reflections	Δρ_max_ = 0.51 e Å^−^^3^
183 parameters	Δρ_min_ = −0.37 e Å^−^^3^

### Special details

**Table:**

Geometry. All esds (except the esd in the dihedral angle between two l.s. planes) are estimated using the full covariance matrix. The cell esds are taken into account individually in the estimation of esds in distances, angles and torsion angles; correlations between esds in cell parameters are only used when they are defined by crystal symmetry. An approximate (isotropic) treatment of cell esds is used for estimating esds involving l.s. planes.

### Fractional atomic coordinates and isotropic or equivalent isotropic displacement parameters (Å^2^)

**Table:**

	*x*	*y*	*z*	*U*_iso_*/*U*_eq_	
Cu1	0.18899 (2)	1.02851 (3)	0.16379 (2)	0.02035 (7)	
I1	0.22857 (2)	1.36381 (2)	0.18629 (2)	0.02246 (6)	
S1	0.01447 (4)	0.97167 (7)	0.10384 (3)	0.01843 (11)	
S2	0.27317 (4)	0.95551 (7)	0.07682 (3)	0.01754 (11)	
O1	−0.12671 (13)	0.4134 (2)	0.18413 (10)	0.0306 (4)	
O2	0.55531 (13)	0.4901 (2)	0.13221 (10)	0.0326 (4)	
N1	−0.12119 (14)	0.6992 (2)	0.15212 (11)	0.0222 (4)	
N2	0.41457 (14)	0.6547 (2)	0.05909 (11)	0.0211 (4)	
C1	−0.09223 (18)	0.6587 (3)	0.08061 (14)	0.0261 (5)	
H1A	−0.1457	0.7049	0.0264	0.031*	
H1B	−0.0897	0.5303	0.0747	0.031*	
C2	0.01160 (17)	0.7356 (3)	0.09339 (15)	0.0237 (5)	
H2A	0.0279	0.7034	0.0437	0.028*	
H2B	0.0656	0.6845	0.1459	0.028*	
C3	−0.03090 (17)	0.9830 (3)	0.18944 (13)	0.0204 (5)	
H3A	0.0220	0.9323	0.2427	0.024*	
H3B	−0.0408	1.1068	0.2011	0.024*	
C4	−0.13193 (17)	0.8852 (3)	0.16610 (14)	0.0226 (5)	
H4A	−0.1559	0.8994	0.2131	0.027*	
H4B	−0.1849	0.9365	0.1130	0.027*	
C5	−0.13845 (16)	0.5668 (3)	0.19871 (13)	0.0227 (5)	
C6	−0.1756 (2)	0.6151 (3)	0.26767 (15)	0.0301 (5)	
H6A	−0.2461	0.6610	0.2406	0.045*	
H6B	−0.1300	0.7045	0.3054	0.045*	
H6C	−0.1748	0.5114	0.3018	0.045*	
C7	0.46423 (17)	0.8128 (3)	0.10528 (14)	0.0241 (5)	
H7A	0.4710	0.8983	0.0640	0.029*	
H7B	0.5345	0.7841	0.1480	0.029*	
C8	0.40226 (17)	0.8939 (3)	0.15130 (14)	0.0244 (5)	
H8A	0.4389	0.9987	0.1833	0.029*	
H8B	0.3973	0.8093	0.1937	0.029*	
C9	0.23472 (16)	0.7451 (3)	0.02483 (13)	0.0195 (4)	
H9A	0.2295	0.6596	0.0668	0.023*	
H9B	0.1658	0.7552	−0.0233	0.023*	
C10	0.31244 (17)	0.6798 (3)	−0.00969 (14)	0.0229 (5)	
H10A	0.2879	0.5677	−0.0403	0.027*	
H10B	0.3172	0.7653	−0.0518	0.027*	
C11	0.46543 (18)	0.4999 (3)	0.07934 (14)	0.0234 (5)	
C12	0.4073 (2)	0.3374 (3)	0.03610 (18)	0.0339 (6)	
H12A	0.4529	0.2358	0.0560	0.051*	
H12B	0.3837	0.3489	−0.0266	0.051*	
H12C	0.3475	0.3221	0.0508	0.051*	

### Atomic displacement parameters (Å^2^)

**Table:**

	*U*^11^	*U*^22^	*U*^33^	*U*^12^	*U*^13^	*U*^23^
Cu1	0.02182 (15)	0.01848 (14)	0.02120 (14)	−0.00092 (11)	0.00916 (12)	−0.00102 (10)
I1	0.03471 (10)	0.01382 (8)	0.01707 (8)	−0.00131 (6)	0.00859 (7)	−0.00134 (5)
S1	0.0196 (3)	0.0185 (3)	0.0176 (2)	0.0000 (2)	0.0080 (2)	0.00071 (19)
S2	0.0196 (3)	0.0148 (2)	0.0179 (2)	−0.0001 (2)	0.0073 (2)	−0.00149 (19)
O1	0.0306 (9)	0.0224 (9)	0.0302 (9)	−0.0033 (7)	0.0034 (7)	0.0003 (7)
O2	0.0305 (10)	0.0403 (10)	0.0256 (9)	0.0143 (8)	0.0099 (8)	0.0080 (7)
N1	0.0240 (10)	0.0211 (10)	0.0236 (9)	−0.0025 (8)	0.0119 (8)	−0.0020 (7)
N2	0.0179 (9)	0.0209 (10)	0.0220 (9)	0.0008 (7)	0.0055 (8)	−0.0049 (7)
C1	0.0296 (13)	0.0257 (12)	0.0245 (12)	−0.0081 (10)	0.0123 (10)	−0.0085 (9)
C2	0.0280 (12)	0.0194 (11)	0.0266 (11)	0.0000 (10)	0.0141 (10)	−0.0055 (9)
C3	0.0245 (12)	0.0203 (11)	0.0184 (10)	0.0004 (9)	0.0108 (9)	−0.0017 (8)
C4	0.0224 (12)	0.0221 (12)	0.0257 (11)	0.0038 (9)	0.0122 (10)	0.0023 (9)
C5	0.0128 (11)	0.0265 (12)	0.0199 (11)	−0.0047 (9)	−0.0025 (9)	0.0005 (9)
C6	0.0292 (13)	0.0332 (14)	0.0290 (12)	−0.0054 (11)	0.0129 (11)	0.0064 (10)
C7	0.0185 (11)	0.0270 (12)	0.0246 (11)	−0.0017 (10)	0.0065 (9)	−0.0059 (9)
C8	0.0189 (12)	0.0299 (12)	0.0203 (11)	0.0004 (10)	0.0036 (9)	−0.0079 (9)
C9	0.0179 (11)	0.0171 (10)	0.0197 (10)	−0.0012 (9)	0.0036 (9)	−0.0021 (8)
C10	0.0206 (11)	0.0246 (11)	0.0197 (11)	0.0009 (9)	0.0042 (9)	−0.0065 (9)
C11	0.0311 (13)	0.0254 (12)	0.0234 (11)	0.0058 (10)	0.0211 (11)	0.0055 (9)
C12	0.0403 (15)	0.0210 (12)	0.0523 (16)	0.0006 (11)	0.0308 (13)	0.0014 (11)

### Geometric parameters (Å, º)

**Table:**

Cu1—S1	2.3012 (6)	C3—H3A	0.9900
Cu1—S2	2.3064 (6)	C3—H3B	0.9900
Cu1—I1	2.6221 (3)	C4—H4A	0.9900
Cu1—I1^i^	2.6476 (3)	C4—H4B	0.9900
I1—Cu1^ii^	2.6476 (3)	C5—C6	1.508 (3)
S1—C3	1.810 (2)	C6—H6A	0.9800
S1—C2	1.815 (2)	C6—H6B	0.9800
S2—C9	1.811 (2)	C6—H6C	0.9800
S2—C8	1.814 (2)	C7—C8	1.521 (3)
O1—C5	1.225 (3)	C7—H7A	0.9900
O2—C11	1.227 (3)	C7—H7B	0.9900
N1—C5	1.366 (3)	C8—H8A	0.9900
N1—C1	1.460 (3)	C8—H8B	0.9900
N1—C4	1.462 (3)	C9—C10	1.523 (3)
N2—C11	1.357 (3)	C9—H9A	0.9900
N2—C10	1.457 (3)	C9—H9B	0.9900
N2—C7	1.459 (3)	C10—H10A	0.9900
C1—C2	1.515 (3)	C10—H10B	0.9900
C1—H1A	0.9900	C11—C12	1.508 (3)
C1—H1B	0.9900	C12—H12A	0.9800
C2—H2A	0.9900	C12—H12B	0.9800
C2—H2B	0.9900	C12—H12C	0.9800
C3—C4	1.518 (3)		
			
S1—Cu1—S2	114.28 (2)	O1—C5—N1	121.6 (2)
S1—Cu1—I1	112.179 (17)	O1—C5—C6	120.6 (2)
S2—Cu1—I1	101.246 (16)	N1—C5—C6	117.7 (2)
S1—Cu1—I1^i^	108.190 (16)	C5—C6—H6A	109.5
S2—Cu1—I1^i^	110.870 (16)	C5—C6—H6B	109.5
I1—Cu1—I1^i^	109.949 (9)	H6A—C6—H6B	109.5
Cu1—I1—Cu1^ii^	126.245 (8)	C5—C6—H6C	109.5
C3—S1—C2	97.16 (10)	H6A—C6—H6C	109.5
C3—S1—Cu1	107.58 (7)	H6B—C6—H6C	109.5
C2—S1—Cu1	102.08 (7)	N2—C7—C8	111.16 (18)
C9—S2—C8	97.32 (10)	N2—C7—H7A	109.4
C9—S2—Cu1	113.27 (7)	C8—C7—H7A	109.4
C8—S2—Cu1	104.68 (7)	N2—C7—H7B	109.4
C5—N1—C1	119.82 (18)	C8—C7—H7B	109.4
C5—N1—C4	125.09 (18)	H7A—C7—H7B	108.0
C1—N1—C4	115.07 (17)	C7—C8—S2	112.12 (15)
C11—N2—C10	124.83 (18)	C7—C8—H8A	109.2
C11—N2—C7	119.84 (18)	S2—C8—H8A	109.2
C10—N2—C7	115.28 (17)	C7—C8—H8B	109.2
N1—C1—C2	112.30 (18)	S2—C8—H8B	109.2
N1—C1—H1A	109.1	H8A—C8—H8B	107.9
C2—C1—H1A	109.1	C10—C9—S2	110.89 (15)
N1—C1—H1B	109.1	C10—C9—H9A	109.5
C2—C1—H1B	109.1	S2—C9—H9A	109.5
H1A—C1—H1B	107.9	C10—C9—H9B	109.5
C1—C2—S1	112.54 (16)	S2—C9—H9B	109.5
C1—C2—H2A	109.1	H9A—C9—H9B	108.0
S1—C2—H2A	109.1	N2—C10—C9	111.90 (17)
C1—C2—H2B	109.1	N2—C10—H10A	109.2
S1—C2—H2B	109.1	C9—C10—H10A	109.2
H2A—C2—H2B	107.8	N2—C10—H10B	109.2
C4—C3—S1	111.67 (14)	C9—C10—H10B	109.2
C4—C3—H3A	109.3	H10A—C10—H10B	107.9
S1—C3—H3A	109.3	O2—C11—N2	121.7 (2)
C4—C3—H3B	109.3	O2—C11—C12	120.4 (2)
S1—C3—H3B	109.3	N2—C11—C12	117.9 (2)
H3A—C3—H3B	107.9	C11—C12—H12A	109.5
N1—C4—C3	111.95 (18)	C11—C12—H12B	109.5
N1—C4—H4A	109.2	H12A—C12—H12B	109.5
C3—C4—H4A	109.2	C11—C12—H12C	109.5
N1—C4—H4B	109.2	H12A—C12—H12C	109.5
C3—C4—H4B	109.2	H12B—C12—H12C	109.5
H4A—C4—H4B	107.9		
			
C5—N1—C1—C2	−120.4 (2)	C11—N2—C7—C8	−120.0 (2)
C4—N1—C1—C2	61.1 (3)	C10—N2—C7—C8	62.5 (2)
N1—C1—C2—S1	−59.4 (2)	N2—C7—C8—S2	−60.5 (2)
C3—S1—C2—C1	52.97 (17)	C9—S2—C8—C7	54.16 (18)
Cu1—S1—C2—C1	162.73 (14)	Cu1—S2—C8—C7	170.60 (15)
C2—S1—C3—C4	−53.85 (17)	C8—S2—C9—C10	−54.05 (16)
Cu1—S1—C3—C4	−158.97 (13)	Cu1—S2—C9—C10	−163.52 (12)
C5—N1—C4—C3	119.1 (2)	C11—N2—C10—C9	118.9 (2)
C1—N1—C4—C3	−62.5 (2)	C7—N2—C10—C9	−63.8 (2)
S1—C3—C4—N1	61.7 (2)	S2—C9—C10—N2	61.7 (2)
C1—N1—C5—O1	2.6 (3)	C10—N2—C11—O2	172.7 (2)
C4—N1—C5—O1	−179.1 (2)	C7—N2—C11—O2	−4.6 (3)
C1—N1—C5—C6	−175.7 (2)	C10—N2—C11—C12	−8.6 (3)
C4—N1—C5—C6	2.6 (3)	C7—N2—C11—C12	174.10 (19)

Symmetry codes: (i) −*x*+1/2, *y*−1/2, −*z*+1/2; (ii) −*x*+1/2, *y*+1/2, −*z*+1/2.

### Hydrogen-bond geometry (Å, º)

**Table:**

*D*—H···*A*	*D*—H	H···*A*	*D*···*A*	*D*—H···*A*
C4—H4*A*···O2^ii^	0.99	2.52	3.241 (3)	129
C6—H6*B*···O2^ii^	0.98	2.47	3.418 (3)	162
C10—H10*A*···O1^iii^	0.99	2.58	3.144 (3)	116
C12—H12*B*···O2^iv^	0.98	2.59	3.372 (3)	137

Symmetry codes: (ii) −*x*+1/2, *y*+1/2, −*z*+1/2; (iii) −*x*, −*y*+1, −*z*; (iv) −*x*+1, −*y*+1, −*z*.
